# Effect of omega-3 supplementation on cardiometabolic indices in diabetic patients with non-alcoholic fatty liver disease: a randomized controlled trial

**DOI:** 10.1186/s40795-021-00490-8

**Published:** 2021-12-15

**Authors:** Abbas Ali Sangouni, Zahra Orang, Hassan Mozaffari-Khosravi

**Affiliations:** 1 Department of Nutrition, School of Public Health, Shahid Sadoughi University of Medical Sciences, Yazd, Iran; 2 Nutrition and Food Security Research Center, School of Public Health, Shahid Sadoughi University of Medical Sciences, Yazd, Iran

**Keywords:** Non-alcoholic fatty liver disease, Diabetes mellitus, Omega-3, Atherogenic, Cardiometabolic

## Abstract

**Background:**

Patients with non-alcoholic fatty liver disease (NAFLD) as well as type 2 diabetes mellitus (T2DM) are at increased risk for cardiovascular diseases (CVD). Omega-3 supplementation has been proposed as a possible strategy for management of cardiometabolic risk. Cardiometabolic indices can predict and evaluate the cardiometabolic risk.

**Aims:**

We investigated the effect of omega-3 supplementation on accurate and available cardiometabolic indices including atherogenic index of plasma (AIP), Castelli risk index I, Castelli risk index II and atherogenic coefficient (AC) in diabetic patients with NAFLD.

**Methods:**

We conducted a double-blind, randomized controlled trial (RCT) for 12 weeks. From August 2016 to March 2017, the subjects referred to Faghihi hospital in Shiraz, Iran, were recruited. Sixty diabetic patients with NAFLD were randomly assigned into the omega-3 (2000 mg/d omega-3 capsule contained 360 mg/d eicosapentaenoic acid and 240 mg/d docosahexaenoic acid) and the placebo (liquid paraffin) groups using computer-generated random number table.

**Results:**

Omega-3 supplementation compared to the placebo had no significant effect on AIP (− 0.11 ± 0.20 vs. -0.03 ± 0.16; *P* = 0.11), Castelli risk index I (− 0.25 ± 0.6 vs. -0.07 ± 0.7; *P* = 0.42), Castelli risk index II (− 0.24 ± 0.5 vs. -0.14 ± 0.5; *P* = 0.63) and AC (− 0.25 ± 0.6 vs. -0.07 ± 0.7; *P* = 0.42). After adjusting for confounding factors, the findings remained without change.

**Conclusion:**

Omega-3 supplementation (2000 mg/d) for 12 weeks has no effect on cardiometabolic risk. It seems, higher doses of omega-3 can improve cordiometabolic risk. The trial was registered at Iranian Registry of Clinical Trials IRCT2016102530489N1.

## Introduction

Non-alcoholic fatty liver disease (NAFLD) encompasses a range of diseases from simple steatosis to non-alcoholic steatohepatitis (NASH) [1, 2]. There is a close relationship between NAFLD and type 2 diabetes mellitus (T2DM) [3]. Insulin resistance, obesity and dyslipidemia are the main factors in the pathogenesis of NAFLD and T2DM [4–6]. The worldwide prevalence of NAFLD is 25.24%, and the prevalence of T2DM in the general population is estimated to be 6.3% [7, 8]. Cardiovascular disease (CVD) is one of the most common complications among patients with T2DM and NAFLD [6, 9]. Cardiometabolic indices such as atherogenic index of plasma (AIP), Castelli risk indices and atherogenic coefficient (AC) are the accurate and available tools that can predict and evaluate cardiometabolic risk [10–12]. Some recent clinical studies have used these indices to evaluate cardiometabolic risk [13, 14]. Total cholesterol (TC), triglyceride (TG), low density lipoprotein-cholesterol (LDL-c) and high density lipoprotein-cholesterol (HDL-c) are used to calculate these cardiometabolic indices [10–12].

It has been suggested that omega-3 polyunsaturated fatty acids (PUFAs), especially eicosapentaenoic acid (C20: 5n3, EPA) and docosahexaenoic acid (C22: 6n3, DHA), can improve insulin resistance, dyslipidemia and obesity have been suggested [15–17]. The experimental studies revealed that PUFAs can increase insulin sensitivity, decrease the production of very low-density lipoprotein (VLDL), and attenuate the accumulation of triglycerides in the liver [18–20]. In addition, PUFAs through modulating endothelium-relaxing, stimulating the production of gasotransmitters leading to vasorelaxation such as nitric oxide (NO) and hydrogen sulphide, and decreasing the production of endothelin 1 and angiotensin II as vasoconstricting factors can improve endothelial dysfunction and atherosclerosis [16, 21]. Some investigations with contradictory results have investigated the effects of omega-3 fatty acids on cardiometabolic outcomes [22–26]. The findings of these studies are inconsistent. In addition, there is no controlled trial evaluating the effect of omega-3 fatty acids on new cardiometabolic indices such as AIP, Castelli risk indices and AC in diabetic patients with NAFLD. Therefore, the present study was designed to investigate the effect of omega-3 fatty acids supplementation on cardiometabolic indices including AIP, Castelli risk index I, Castelli risk index II and AC in diabetic patients with NAFLD.

## Methods

### Recruitment and eligibility screening

From August 2016 to March 2017, subjects referred to the Faghihi hospital in Shiraz, Iran, were recruited and screened. Sixty patients met the inclusion criteria. The inclusion criteria were: age 18 to 65 years, diagnosis of T2DM according to the American Diabetes Association (ADA) criteria [27], and diagnosis of NAFLD by a gastroenterologist using ultrasonography (grades 1, 2 and 3). The exclusion criteria were: kidney diseases, type 1 diabetes, thyroid diseases, viral hepatitis, cancers, alcohol consumption, insulin infusion, pregnancy, allergy to seafood, adherence to a specific diet, poor compliance and unwillingness to continue the study.

### Trial design

The present double-blind, randomized controlled trial (RCT) was conducted for 12 weeks. The participants signed a written informed consent approved by the ethical committee of Shahid Sadoughi University of Medical Sciences and Health Services, Yazd (IR.SSU.SPH.REC.1395.67). The trial was registered on 06/02/2017 at the Iranian clinical trials website under code number: IRCT2016102530489N1. The random allocation and assignment of participants into the treatment and the control groups was performed by a trained person who was not involved in the trial. Group allocation was concealed from patients and investigators. Simple (unrestricted) randomization was performed using a computer-generated random number table that was produced by a random allocation software [28]. The participants and investigators were blinded until the end of the trial.

### Intervention

All omega-3 and placebo capsules were manufactured by Zahravi Pharmaceutical Company, Tabriz, Iran. The treatment group received 1000 mg omega-3 as gel capsule twice per day (each 1000 mg capsule contained 180 mg EPA and 120 mg DHA), and the control group received the same amount of placebo (liquid paraffin) in the form of gel capsule. At the time we designed our original study, there was little evidence about the appropriate and safe dose of omega-3. The dose of omega-3 in the study of Samimi et al. [29] was 1000 mg/d (one capsule containing 180 mg/d EPA and 120 mg/d DHA). This study reported that 1000 mg/d omega 3 can improve insulin, but has no effect on lipid profile [29]. This study did not report serious adverse event related to the dosage of omega-3 [29]. We selected higher dose of omega-3 (2000 mg/d) and longer intervention duration (12 weeks) than the study of Samimi et al. [29]. The participants were asked to consume one capsule an hour before lunch and one capsule an hour before dinner. The appearance of omega-3 capsules was similar to placebo capsules. Labeling the supplement boxes was performed as A or B by a third person. The capsules were given to the participants each month. To evaluate the compliance rate, the participants were asked to deliver empty boxes of capsules to the researcher. Consuming less than 80% of expected amounts of capsules was considered the poor compliance.

### Dietary intake and physical activity assessment

At the baseline and the end of the trial, the average intake of energy as well as macronutrients was measured using a 3-day 24-h recall questionnaire. We used Nutritionist IV software (version 7.0; N-Squared Computing, Salem, OR, USA), modified for Iranian foods, to calculate intake of energy and macronutrients [30].

Physical activity can independently improve various health outcomes. In other words, significant difference between groups in physical activity can distort the real effect of intervention on outcomes. Therefore, the metabolic equivalent of task (MET) questionnaire [31] was used to assess the level of physical activity at the baseline and the end of the study.

### Laboratory and anthropometric evaluations

TC, TG, LDL-c and HDL-c concentrations were measured at the baseline and the weeks 12. After 10 h fasting, 10 ml blood was collected and centrifuged. Serums were immediately frozen at − 80 °C. Levels of TC, TG, LDL-c and HDL-c were measured by an autoanalyzer (AVIDA 1800 chemistry system; Siemens, United Kingdom) using Pars Azmoon, Iran kits.

### Anthropometric evaluations

Height, weight, and waist circumference (WC) were measured at the baseline and the end of the study. Assessment of height and WC was performed under the standard protocols by a measuring tape. Weight was measured by a scale (Omeron, Japan) with an accuracy of 100 g, while the participants were with light clothes and without shoes. Body mass index (BMI) was calculated by the following formula: weight (kg)/height squared (m^2^).

### Cardiometabolic indices

AIP [11], Castelli risk index I [10], Castelli risk index II [10] and AC [12] were calculated at baseline and at the end of the trial by the following equations:

AIP = log (TG/HDL − c) [11].

Castelli risk index I = TC/HDL − c [10].

Castelli risk index II = LDL − c/HDL − c [10].

AC = (TC − HDL − c)/HDL − c [12].

### Sample size and statistical analysis

In our previous article that reported the findings of insulin resistance, body composition and lipid profile [26], the sample size was estimated to be 30 for each group with 95% confidence interval, α = 0.05, power = 80%. The power analysis was performed again, and we estimated power = 80% for this article. Analysis of data was done using the Statistical Package for Social Science (SPSS) software (Chicago, Illinois, USA) version 24. To evaluate the normal distribution of variables, the Kolmogorov–Smirnov test was used. The intention-to-treat (ITT) approach was used. To compare the general characteristics between groups, we used independent t-test and chi-square test for quantitative and qualitative variables, respectively. To compare means of variables in each group, paired t-test was used. In addition, the univariate ANCOVA was used to eliminate the effects of confounding factors. *P*-values less than 0.05 were considered the significant level.

## Results

### Characteristics of the participants

**Fig. 1:**
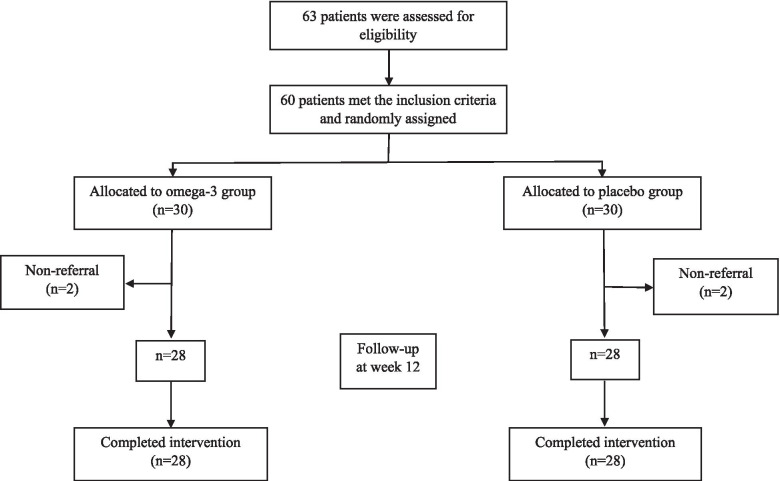
Flowchart of eligibility, screening and follow-up

Sixty participants were included and with a ratio of 1:1 were randomly assigned into the treatment and the control groups. Four participants were excluded from the follow-up due to non-referral. Fifty-six participants including 28 patients in the treatment group and 28 patients in the control group completed the study (Fig. 1). At the baseline, no significant difference between the two groups was observed in baseline characteristics (*P* > 0.05); however, the intake of energy in the treatment group was significantly higher compared to the control group (*P* = 0.002) (Table 1).

**Table 1:** Baseline characteristics in diabetic patients with NAFLD

	Omega-3 (***n*** = 30)	Placebo (***n*** = 30)
Age*, y	48.6 ± 7.6	48.8 ± 8.7
Gender*		
Male, n (%)	9 (30)	6 (20)
Female, n (%)	21 (70)	24 (80)
**Energy intake**, kcal/d	1585.1 ± 572.8	1200.2 ± 295.5
**MET**, (h/d)	28.1 ± 4.2	27.7 ± 3.9
Height*, cm	160.4 ± 8.9	160.5 ± 8.2
Weight*, kg	78.0 ± 10.5	77.1 ± 12.5
BMI*, kg/m^2^	30.2 ± 3.6	29.8 ± 4.2
WC*, cm	106.7 ± 9.1	104.2 ± 9.8
HDL-c*, mg/dL	45.3 ± 8.9	46.5 ± 8.03
LDL-c*, mg/dL	95.9 ± 30.0	92.2 ± 22.2
TG*, mg/dL	151.6 ± 57.5	138.4 ± 51.8
TC*, mg/dL	166.0 ± 41.1	159.9 ± 29.3
AIP*	0.49 ± 0.22	0.44 ± 0.19
Castelli risk index I*	3.63 ± 0.9	3.41 ± 0.7
Castelli risk index II*	2.11 ± 0.6	1.97 ± 0.5
AC*	2.63 ± 0.9	2.41 ± 0.7

*P* values are computed by independent t-test and data are expressed as mean ± standard deviation (SD), while for Gender is computed by chi-square test and data are expressed as number (percent)

*: No significant difference was found between two groups at the baseline, except for energy intake (*P* = 0.002)

*NAFLD* Non-alcoholic fatty liver disease, *MET-h* Metabolic equivalent task, *BMI* Body mass index, *WC* Waist circumference, *HDL-c* High density lipoprotein-cholesterol, *TG* Triglyceride, *TC* Total cholesterol, *AIP* Atherogenic index of plasma, *AC* Atherogenic coefficient

No significant difference was found between the two groups in the terms of weight (*P* = 0.61), BMI (*P* = 0.56) and WC (*P* = 0.27) (Table 2). Participants did not report any serious adverse effect during intervention.

**Table 2:** Anthropometric variables in diabetic patients with NAFLD

Anthropometric variables	Omega-3 (n = 30)	Placebo (n = 30)	***P***	***P***^†^
**Weight**, kg			0.71	0.61
Baseline	78.0 ± 10.5	77.1 ± 12.5		
Week 12	78.0 ± 11.1	76.8 ± 13.8		
Mean change	0.0 ± 2.3	− 0.3 ± 2.3		
**BMI**, kg/m^2^			0.59	0.56
Baseline	30.2 ± 3.6	29.8 ± 4.2		
Week 12	30.2 ± 3.7	29.6 ± 4.5		
Mean change	0.0 ± 0.8	−0.2 ± 0.8		
**WC**, cm			0.59	0.27
Baseline	106.7 ± 9.1	104.2 ± 9.8		
Week 12	106.1 ± 8.2	104.8 ± 9.6		
Mean change	−0.7 ± 3.7	0.6 ± 3.2		

*P* values are computed by independent t-test and data are expressed as mean ± standard deviation (SD)

*P*: resulted from comparing the means of each variable at the end of the study, between two groups

*P*^†^: resulted from comparing the mean change from baseline between groups

*NAFLD* Non-alcoholic fatty liver disease, *BMI* Body mass index, *WC* Waist circumference

### Outcomes

No significant difference between the treatment group and the control group was found in the terms of AIP (0.49 ± 0.22 vs. 0.44 ± 0.19; *P* = 0.36), Castelli risk index I (3.63 ± 0.9 vs. 3.41 ± 0.7; *P* = 0.31), Castelli risk index II (2.11 ± 0.6 vs. 1.97 ± 0.5; *P* = 0.55) and AC (2.63 ± 0.9 vs. 2.41 ± 0.7; *P* = 0.31) at the baseline. In addition, there was no significant difference between the treatment and control groups in AIP (0.38 ± 0.28 vs. 0.41 ± 0.27; *P* = 0.10), Castelli risk index I (3.38 ± 0.9 vs. 3.34 ± 0.7; *P* = 0.29), Castelli risk index II (1.87 ± 0.6 vs. 1.83 ± 0.5; *P* = 0.47) and AC (2.38 ± 0.9 vs. 2.34 ± 0.7; *P* = 0.29) at the end of the trial (Table 3).

**Table 3:** Effect of omega-3 on cardiometabolic indices in diabetic patients with NAFLD

Indices	Omega-3 (n = 30)	Placebo (n = 30)	***P***^†^	***P***^††^
**AIP**				0.13
Baseline	0.49 ± 0.22	0.44 ± 0.19	0.36	
Week 12	0.38 ± 0.28	0.41 ± 0.27	0.67	
***P***	0.007	0.33		
Mean change	−0.11 ± 0.20	−0.03 ± 0.16	0.10	
**Castelli risk index I**				0.46
Baseline	3.63 ± 0.9	3.41 ± 0.7	0.31	
Week 12	3.38 ± 0.9	3.34 ± 0.7	0.88	
***P***	0.05	0.62		
Mean change	−0.25 ± 0.6	−0.07 ± 0.7	0.29	
**Castelli risk index II**				0.70
Baseline	2.11 ± 0.6	1.97 ± 0.5	0.55	
Week 12	1.87 ± 0.6	1.83 ± 0.5	0.81	
***P***	0.01	0.15		
Mean change	−0.24 ± 0.5	−0.14 ± 0.5	0.47	
**AC**				0.40
Baseline	2.63 ± 0.9	2.41 ± 0.7	0.31	
Week 12	2.38 ± 0.9	2.34 ± 0.7	0.88	
***P***	0.05	0.62		
Mean change	−0.25 ± 0.6	−0.07 ± 0.7	0.29	

*P* values are computed by independent t-test and data are expressed as mean ± standard deviation (SD)

*P*: resulted from comparisons within groups by paired t-test

*P*^†^: resulted from comparisons between two groups by independent t-test

*P*^††^: resulted from comparing the mean change from baseline between groups using univariate ANCOVA after adjusting for energy intake and weight change

*NAFLD* Non-alcoholic fatty liver disease, *AIP* Atherogenic index of plasma, *AC* Atherogenic coefficient

According to mean changes, 12-week omega-3 supplementation compared to the placebo had no significant effect on AIP (− 0.11 ± 0.20 vs. -0.03 ± 0.16; *P* = 0.11), Castelli risk index I (− 0.25 ± 0.6 vs. -0.07 ± 0.7; *P* = 0.42) and Castelli risk index II (− 0.24 ± 0.5 vs. -0.14 ± 0.5; *P* = 0.63) as well as AC (− 0.25 ± 0.6 vs. -0.07 ± 0.7; *P* = 0.42) (Table 3). After adjusting for confounding factors, the findings remained without change.

## Discussion

We demonstrated that 2000 mg/d omega-3 supplementation for 12 weeks has no effect on cardiometabolic indices including AIP, Castelli risk index I, Castelli risk index II and AC. The AIP, AC and Castelli risk indices are the accurate and available tools to assess the cardiometabolic risk [11, 12, 32, 33], and there is a direct correlate between the levels of these indices and cardiometabolic risks [11, 12, 33]. To our knowledge, the present study was the first RCT investigating the effect of omega-3 supplementation on atherogenic and Castelli risk indices in diabetic patients with NAFLD. In a randomized open-labeled trial that was conducted by Zibaeenezhad et al. [34], 2 g/d omega-3 supplementation for 8 weeks had no effect on AIP and Castelli risk indices; but, fresh fish consumption could improve these indices. In addition to omega-3 fatty acids, fresh fish has several nutrients such as selenium, vitamin D and antioxidants [35, 36]. These nutrients altogether can reduce lipid peroxidation, myocardial infarct size as well as ischemia-induced ventricular arrhythmias, and improve recovery from ischemia [35, 36]. This can explain the greater effects of fresh fish compared to the omega-3 supplement on cardiometabolic risk. Some investigations have suggested that omega-3 fatty acids can increase production of NO and hydrogen sulphide, reduce the production of vasoconstrictors such as endothelin 1 and angiotensin II, and subsequent can improve the endothelial dysfunction, reduce the platelet aggregation and blood pressure [16, 21, 37, 38]. In addition, omega-3 fatty acids through decreasing gene expression of fatty acid synthase (FAS), and phosphoenolpyruvate carboxykinase (PEPCK), reducing insulin resistance and inhibiting the inflammatory pathways triggered by cytokines can improve cardiometabolic risk [17, 21, 38–40]. The main parts of atherogenic and Castelli risk indices are serum levels of TC, TG, LDL-c and HDL-c [11, 12, 33]. Several investigations have examined the effect of omega-3 fatty acids on these variables. The study of Dasarathy et al. [24] reported that omega-3 supplementation has no effect on serum concentrations of TC, LDL-c and HDL-c. In addition, our original study demonstrated that omega-3 supplementation for 12 weeks reduced serum TG levels, but could not improve serum concentrations of TC, LDL-c and HDL-c in diabetic patients with NAFLD [26]. Moreover, the study of Scorletti et al. [41] demonstrated that after intervention for 15–18 months, there is no significant difference between the omega-3 group (4 g/d) and the placebo group in levels of TC, LDL-c and HDL-c. However, the study of Zhu et al. [42] showed a significant reduction in serum LDL-c, TG after omega-3 supplementation (6 g/d) for 24 weeks in patients with NAFLD, but the serum levels of TC and HDL-c remained without significant change. A meta-analysis on RCTs in patients with NAFLD revealed that omega-3 supplementation can reduce TG [43]. It seems, 2–4 g/d omega-3 has a beneficial effect on TG, but not TC, LDL-c and HDL-c. Omega-3 by inducing β-oxidation, decreasing VLDL synthesis, regulating gene expression of sterol-regulatory element-binding protein-1 (SREBP-1) and carbohydrate response element binding protein (ChREBP), modulating the TG synthesis, activating phosphatidic acid, diacylglycerol acyltransferase as well as LPL, and reducing the TG clearance from circulating VLDL particles can regulate serum TG [17, 21, 44–46]. To follow the principals of ethics in research, we clarify that our research group reported the effect of omega-3 fatty acids on fatty liver, visceral adiposity index, insulin resistance, body composition and lipid profile, previously [26, 47]. We used the same data for the present article. The flowchart of eligibility, screening and follow-up as figure as well as some necessary information as table and sample size information of our previous article [26] were added to the present article.

An important strength of the present study, we measured the important confounding factors such as anthropometric variables during the follow-up. The present trial has some important limitations. We used liver ultrasonography due to its noninvasiveness and availability. Fibroscan has higher accuracy than ultrasonography in assessing severity of NAFLD [48, 49]. Based on ultrasonography, it was possible that occur a false negative diagnosis in sampling of patients with NAFLD; however, the possible of false positive diagnosis was very low [48, 50]. As another important limitation, we did not use cutoff point for alanine aminotransferase (ALT) levels in inclusion criteria. In addition, we used low dose of omega-3, and this is another important limitation of this study.

## Conclusion

Omega-3 supplementation (2000 mg/d) has no effect on cardiometabolic indices such as AIP, Castelli risk index I, Castelli risk index II and AC. Further studies with longer intervention durations and higher doses of omega-3 are still required to clarify our vision.

## Data Availability

The datasets used and/or analyzed during the current study available from the corresponding author on reasonable request.

## Abbreviations

AC: Atherogenic coefficient
ADA: American Diabetes Association
AIP: Atherogenic index of plasma
ALT: Alanine aminotransferase
BMI: Body mass index
ChREBP: Carbohydrate-responsive element-binding protein
DHA: Docosahexaenoic acid
EPA: Eicosapentaenoic acid
FAS: Fatty acid synthase
HDL-c: High-density lipoprotein cholesterol
ITT: Intention-to-treat
LDL-c: Low-density lipoprotein cholesterol
LPL: Lipoprotein lipase
NAFLD: Non-alcoholic fatty liver disease
MET: Metabolic equivalent of task
PEPCK: Phosphoenolpyruvate carboxykinase
RCT: Randomized controlled trial
SPSS: Statistical Package for Social Science
SREBP-1: Sterol-regulatory element-binding protein-1
No: Nitric oxide
T2DM: Type 2 diabetes mellitus
TC: Total cholesterol
TG: Triglyceride
VAI: Visceral adiposity index
VLDL: Very low-density lipoprotein
WC: Waist circumference
